# Regulation of the microvasculature during small muscle mass exercise in chronic obstructive pulmonary disease vs. chronic heart failure

**DOI:** 10.3389/fphys.2022.979359

**Published:** 2022-08-31

**Authors:** Jacob Peter Hartmann, Rasmus H. Dahl, Stine Nymand, Gregers W. Munch, Camilla K. Ryrsø, Bente K. Pedersen, Pia Thaning, Stefan P. Mortensen, Ronan M. G. Berg, Ulrik Winning Iepsen

**Affiliations:** ^1^ Centre for Physical Activity Research, Copenhagen University Hospital, Rigshospitalet, Copenhagen, Denmark; ^2^ Department of Clinical Physiology and Nuclear Medicine, Copenhagen University Hospital, Rigshospitalet, Copenhagen, Denmark; ^3^ Department of Radiology, Hvidovre Hospital, Copenhagen, Denmark; ^4^ Department of Radiology, University Hospital Rigshospitalet, Copenhagen, Denmark; ^5^ Department of Biomedical Sciences, Faculty of Health and Medical Sciences, University of Copenhagen, Copenhagen, Denmark; ^6^ Department of Pulmonary and Infectious Diseases, Copenhagen University Hospital, North Zealand, Hillerød, Denmark; ^7^ Department of Respiratory Medicine, Copenhagen University Hospital, Hvidovre Hospital, Copenhagen, Denmark; ^8^ Department of Cardiovascular and Renal Research, University of Southern Denmark, Copenhagen, Denmark; ^9^ Neurovascular Research Laboratory, Faculty of Life Sciences and Education, University of South Wales, Pontypridd, United Kingdom; ^10^ Department of Anaesthesiology and Intensive Care, Copenhagen University Hospital, Bispebjerg Hospital, Copenhagen, Denmark

**Keywords:** COPD, chronic obstructive pulmonary disease, heart failiure, knee extensor exercise, capillary recruitment, exercise capacity

## Abstract

**Aim:** Skeletal muscle convective and diffusive oxygen (O_2_) transport are peripheral determinants of exercise capacity in both patients with chronic obstructive pulmonary disease (COPD) and chronic heart failure (CHF). We hypothesised that differences in these peripheral determinants of performance between COPD and CHF patients are revealed during small muscle mass exercise, where the cardiorespiratory limitations to exercise are diminished.

**Methods:** Eight patients with moderate to severe COPD, eight patients with CHF (NYHA II), and eight age- and sex-matched controls were studied. We measured leg blood flow (Q̇_leg_) by Doppler ultrasound during submaximal one-legged knee-extensor exercise (KEE), while sampling arterio-venous variables across the leg. The capillary oxyhaemoglobin dissociation curve was reconstructed from paired femoral arterial-venous oxygen tensions and saturations, which enabled the estimation of O_2_ parameters at the microvascular level within skeletal muscle, so that skeletal muscle oxygen conductance (D_SM_O_2_) could be calculated and adjusted for flow (D_SM_O_2_/Q̇_leg_) to distinguish convective from diffusive oxygen transport.

**Results:** During KEE, Q̇_leg_ increased to a similar extent in CHF (2.0 (0.4) L/min) and controls (2.3 (0.3) L/min), but less in COPD patients (1.8 (0.3) L/min) (*p* <0.03). There was no difference in resting D_SM_O_2_ between COPD and CHF and when adjusting for flow, the D_SM_O_2_ was higher in both groups compared to controls (COPD: 0.97 (0.23) vs. controls 0.63 (0.24) mM/kPa, *p*= 0.02; CHF 0.98 (0.11) mM/kPa vs. controls, *p*= 0.001). The Q̇-adjusted D_SM_O_2_ was not different in COPD and CHF during KEE (COPD: 1.19 (0.11) vs. CHF: 1.00 (0.18) mM/kPa; *p*= 0.24) but higher in COPD vs. controls: 0.87 (0.28) mM/kPa (*p*= 0.02), and only CHF did not increase Q̇-adjusted D_SM_O_2_ from rest (*p*= 0.2).

**Conclusion:** Disease-specific factors may play a role in peripheral exercise limitation in patients with COPD compared with CHF. Thus, low convective O_2_ transport to contracting muscle seemed to predominate in COPD, whereas muscle diffusive O_2_ transport was unresponsive in CHF.

## Introduction

Exercise intolerance is a cardinal feature of both chronic obstructive pulmonary disease (COPD) and chronic heart failure (CHF) (Vestbo et al., 2013; Buber and Robertson, 2022). The associated decline in peak oxygen uptake (V̇O_2_peak) during whole-body exercise in these disease states has been attributed to ‘central’ factors, i.e., cardio-pulmonary limitations, but emerging evidence suggests that peripheral factors, such as limb muscle dysfunction, also contributes to the exercise intolerance (Maltais et al., 2014; Keller-Ross et al., 2019). The limb muscle dysfunction, characterised by a low oxidative enzyme capacity, fibre type shift (type I to II) and muscle wasting, is quite similar in COPD and CHF (Zizola and Schulze, 2013; Ausín et al., 2017). Thus, studying how the skeletal muscle maladapts to chronic pulmonary or cardiac disease may enhance our understanding of the peripheral contribution to exercise intolerance in these common disorders.

The diminished V̇O_2_peak in both COPD and CHF during whole-body exercise may be explained by any limiting factor of the integrated O_2_ transport system, including ventilation, alveolar-to-capillary diffusion, cardiac output, blood volume, muscle blood supply and muscle diffusion (Wagner, 1996; Houstis et al., 2018). Whole body V̇O_2_peak testing provoke cardio-respiratory symptoms in COPD and CHF due to the large muscle mass activated, thus masking any peripheral skeletal muscle limitations (Mortensen and Saltin, 2014; Poole et al., 2018; Dempsey, 2019). Small muscle mass exercise using one-legged knee-extensor exercise (KEE) is an established method to determine peripheral limitations to V̇O_2_peak in COPD and CHF, because both cardiac output and minute ventilation are affected to a lesser degree compared to whole body exercise (Mortensen et al., 2010). Importantly, a higher skeletal muscle peak oxygen uptake (V̇O_2SM_peak) may be obtained during KEE compared to whole-body exercise in these patients (Richardson et al., 2004; Barrett-O’Keefe et al., 2014).

Oxygen transport to skeletal muscle determines peripheral exercise capacity, which depends on the convective delivery of blood flow (Q̇) to the capillary bed and diffusive transport from red blood cells to myocyte mitochondria (Poole et al., 2022). Blood flow to contracting muscle is tightly regulated to match oxygen delivery to metabolic demand in healthy humans (Saltin, 1985; Mortensen and Saltin, 2014). The pulmonary diffusion deficits in COPD or reduced cardiac output in CHF are likely to impact regulation of muscle convective and diffusive O_2_ transport in response to exercise, as both diseases share limb muscle maladaptions abnormalities. Notwithstanding, the neural and metabolic interactions, that may also contribute to the peripheral exercise intolerance (Gagnon et al., 2012; Maltais et al., 2014; Smith et al., 2020). Indeed, previous findings suggest that both peripheral muscle O_2_ convective delivery (Brønstad et al., 2012) and diffusive O_2_ transport are impaired during maximal KEE in COPD (Broxterman et al., 2021), while muscle convective O_2_ delivery has been shown to be attenuated in CHF (Barrett-O’Keefe et al., 2014, 2019).

As of now, no studies have directly compared the peripheral limitations of the oxygen transport cascade in these predominantly centrally limited disease states to determine whether disease-specific microvascular (mal)adaptations are present. In the present study, we hypothesised that such disease-specific differences between the COPD and CHF patients are present, with COPD showing both convective as well as diffusive limitation whereas CHF are mainly limited on their convective capacity compared to controls.

## Materials and methods

### Ethical approval

The studies were approved by the Ethical Committee of the Capital Region of Copenhagen (H-2–2013–150; H-3–2013–048) and performed according to the guidelines of the Declaration of Helsinki. All subjects provided oral and written informed consent prior to enrolment. Experiments were performed in the same laboratory as a part of a larger study and some results have previously been published (Iepsen et al., 2017; Munch et al., 2018). The present study involves novel analyses of arterio-venous blood gases in response to KEE from the two studies to address an independent working hypothesis.

### Participants

We included eight COPD patients (Global Initiative for Chronic Obstructive Lung Disease (GOLD) II and III), eight patients with heart failure with reduced ejection fraction (HFrEF) (New York Heart Association Classification (NYHA) class II), and eight healthy age- and sex-matched controls (Table 1). In the previous study by Iepsen et al., 10 COPD patients were included, but due to technical issues with placement of the arterial catheters, two could not participate in the invasive part of the study (Iepsen et al., 2017). In the study by Munch et al. (Munch et al., 2018), eight healthy controls were included, but two of them did not want to participate in the training intervention and were only enrolled in the cross-sectional study. Due to the invasive nature of this experiment, the control group was the same for both studies. Thus, we studied 8 COPD patients, 8 CHF patients and 8 healthy controls unexposed to exercise training. In one of the COPD patients, we could only calculate arterial blood gas values at rest, and therefore, we excluded this subject from the exercise analyses.

**TABLE 1:** Subject characteristics.

Baseline	COPD	CHF	Control
Age (years)	63 (8)	57 (10)	64 (7)
Men/women	6/2	7/1	7/1
BMI	26 (4)	29 (6)	25 (3)
BP systolic (mmHg)	142 (10)	144 (16)	136 (11)
BP diastolic (mmHg)	87 (7)	85 (7)	85 (9)
Watt max one leg (W)	36 (12)	34 (17)	41 (11)
6MWT (m)	605 (64) #	549 (99) #	663 (47)
V̇O_2peak_ (ml/min)	1819 (358) #	2129 (409)	2575 (549)

Data in means (standard deviation). We performed a one-way ANOVA, and if significant, a Tukey’s honest significant difference was used to analyse the differences between groups. # Different from control group (*p* <0.05). n = 8 in all groups. Abbreviations: COPD: chronic obstructive pulmonary disease. CHF: Chronic heart failure. BMI: body mass index. BP: blood pressure. 6MWT: 6 min walking test. V̇O_2peak_: Peak oxygen consumption.

Inclusion criteria for COPD patients were a forced expiratory volume in 1 s (FEV_1_)/forced vital capacity ratio <0.70, FEV_1_ ≤ 70% of predicted, modified Medical Research Council Dyspnoea Scale ≤2, resting arterial oxygenation >90%, and age 40–80 years. Eligibility criteria for CHF patients were left ventricle ejection fraction <40%, NYHA class < eller lig II and age 40–80 years. All patients were clinically stable and primarily recruited from outpatient clinics. Exclusion criteria were use of anticoagulant medications, diabetes, hypertension, claudication, heart failure (COPD and controls) COPD (CHF and controls), unstable ischemic heart disease or malignant diseases. Sitting spirometry (Model 2120, Vitalograph Ltd. Buckingham, United Kingdom) in COPD, standard echocardiography (Vivid 9, GE Healthcare, Pittsburgh, PA) in CHF, and a general medical examination including blood testing prior to inclusion.

### Experimental procedures

Prior to the experimental day all subjects visited the laboratory to be familiarised to the KEE-model, and performed; (1) an incremental bicycle test on a bicycle ergometer (Monark 839E; Monark, Varberg, Sweden) to determine whole body V̇_O2peak_ (l/min,) and peak workload (Watt_peak_) while oxygen consumption was measured breath-by-breath using a Quark gas analyser (Cosmed, Italy) (2) an incremental KEE test, to determine peak workload, (3) 6 minutes walking distance (6MWD) and (4) whole-body dual-energy X-ray absorptiometry scanning (Lunar Prodigy Advance; GE Healthcare, Madison, WI).

On the experimental day participants refrained from caffeine, alcohol, and exercise for 24 h. The COPD patients paused their medications for 24–48 h depending on the drug profile to avoid potential vasoconstrictor effects of inhaled β_2_-agonists and muscarinic antagonists. The CHF patients paused their ACE-inhibitors 48 h prior to the experimental day, whereas beta-blockers, anti-platelet and diuretic drugs were withdrawn 24 h prior to the experimental day. After local anaesthesia (lidocaine 2%), catheters were placed in the femoral artery and vein of the experimental leg and one in the brachial artery.

KEE at 10 W was performed by all subjects using the same protocol. Prior to the acute exercise bout, the experimental leg was moved passively by an investigator at 60 kicks/min and subjects were instructed to keep that pace during exercise. Leg haemodynamics were evaluated, and arterio-venous blood samples were collected at baseline and during active knee-extensions at steady state (3.5 min of active contractions). Common femoral arterial blood flow was measured by the same sonographer using Doppler ultrasound (Logic E9, GE Healthcare, Milwaukee, WI, United States) equipped with a linear probe (9 MHz), as previously described (Iepsen et al., 2017; Munch et al., 2018). In brief, the site of the blood flow measurements was below the inguinal ligament but well above the bifurcation of the artery and recordings were obtained at the lowest possible insonation angle and always below 60˚. The sample volume was maximised according to the width of the vessel and kept clear of the walls. Arterial diameter was measured during systole from resting arterial B-mode images with the transducer parallel to the vessel. Doppler tracings were averaged over eight heart cycles at the same time as the blood sampling. Blood samples were drawn simultaneous from the femoral venous and arterial catheters and blood gases were immediately analysed (ABL 725, Radiometer, Glostrup, DK).

### Calculations

Oxygen content (C_x_O_2_) was calculated as:

$$CxO_{2}=Hb\cdot SxO_{2}+\alpha O_{2}\cdot PxO_{2}\;$$ (1)

Where S is the oxygen saturation of haemoglobin (fraction) and P is the partial pressure of oxygen (kPa). x denotes either arterial (a), femoral-venous (v), or capillary (cap) blood, Hb ( ${\text{mmol L}}^{-1}$ ) is the haemoglobin concentration and α is the solubility coefficient of oxygen in blood (0.01 ${\text{mmol L}}^{-1}{\text{ kPa}}^{-1}$ ). Oxygen delivery (DelO_2_) to and the fractional oxygen extraction (EO_2_) by skeletal muscle were calculated as:

$$DelO_{2}=CaO_{2}\cdot {\dot{Q}}_{leg}\;$$ (2)

$$EO_{2}=\;\frac{a-vDO_{2}}{CaO_{2}}$$ (3)

where avDO_2_ is the arterial-to-femoral venous oxygen content difference.

Skeletal muscle oxygen uptake ( $\dot{V}O_{2SM}$ ) was calculated as:

$$\dot{V}O_{2SM}=avDO_{2}\cdot \;{\dot{Q}}_{leg}$$ (4)

Leg vascular conductance (LVC) was calculated as:

$$LVC=\;\frac{{\dot{Q}}_{leg}}{MAP}$$ (5)

Where MAP is mean arterial blood pressure (=1/3 systolic blood pressure +2/3 diastolic blood pressure) or area under the curve (AUC) over 8 heart cycles for invasive experiments with pulse contour analysis.

Lastly, skeletal muscle oxygen conductance (D_SM_O_2_) was calculated as:

$$D_{SM}O_{2}=\;\frac{\dot{V}O_{2SM}}{\bar{P}capO_{2}}$$ (6)

where $\bar{P}capO_{2}$ is the mean capillary oxygen tension (see below). Given that $D_{SM}O_{2}$ has both a convective and a diffusive component, it was adjusted for ${\dot{Q}}_{leg}$ by division to provide a surrogate measure of the non-convective, i.e. the diffusive, component:

$$\dot{Q}-\text{adjusted }D_{SM}O_{2}=\;\frac{avDO_{2}}{\bar{P}capO_{2}}$$ (7)

Given that i) the oxygen dissociation curve (ODC) is gradually modulated during the red blood cells’ passage though the microcirculation, e.g., due to changes in pH, PaCO_2_ and temperature, and ii) that the OCD may differ between individual capillaries throughout the skeletal muscle tissue, an ideal ‘averaged’ capillary ODC was modelled using paired arterial and femoral-venous blood gas values. For simplicity, and in accordance with our previous study (Dahl et al., 2020), ‘capillary’ here refers to the part of the microvasculature where gas exchange between blood and tissue takes place. However, it should be kept in mind, that this is not a well-defined anatomical entity, since gas exchange also takes place in other parts of the microvasculature, notably upstream and less so downstream of the anatomically defined capillary bed (Pittman, 2000; Shibata et al., 2006). Thus, it was assumed that measured blood gas values equal the oxygen tension and saturation in the arterial inlet and venous outlet of the gas exchanging microvasculature.

This averaged OCD was defined by the capillary *P*_50_ (*P*_50cap_), which reflects the dissociation constant of haemoglobin, and the capillary Hill coefficient, *h*_*cap*_, which is the cooperativity. A pair of *P*_50cap_- and *h*_*cap*_-values was calculated that fulfils the Hill equation in both arterial and femoral-venous blood (Dahl et al., 2020):

$$h_{cap}=\frac{\text{ln}\left(\frac{SaO_{2}}{1-SaO_{2}}\right)-\text{ln}\left(\frac{SvO_{2}}{1-SvO_{2}}\right)}{\ln\left(PaO_{2}\right)-\ln\left(PvO_{2}\right)}$$ (8)

*P*_50cap_ was then calculated by insertion of the Hill coefficient into the Hill equation using arterial or femoral-venous blood gas values.

$$P_{50cap}=PaO_{2}\cdot {\left(\frac{SaO_{2}}{1-SaO_{2}}\right)}^{-\frac{1}{h_{cap}}}=PvO_{2}\cdot {\left(\frac{SvO_{2}}{1-SvO_{2}}\right)}^{-\frac{1}{h_{cap}}}$$ (9)

The mean capillary oxygen tension ( $\left(\bar{P}capO_{2}\right)$ and saturation in the skeletal muscle microvasculature was determined based on similar principles to those previously outlined for the cerebral microcirculation (Dahl et al., 2020). Notably, the total capillary oxygen content ( $CcapO_{2})$ is assumed to decrease proportionally with the distance travelled as blood flows through the capillary from the arterial inlet to the venous outlet. Formulas for calculation of these parameter are given below:

$$\bar{P}capO_{2}=PvO_{2}+\left(PaO_{2}-PvO_{2}\right)\cdot \frac{CaO_{2}-C\prime }{CaO_{2}-CvO_{2}}$$ (10)

$$\bar{S}capO_{2}=SvO_{2}+\left(SaO_{2}-SvO_{2}\right)\cdot \frac{CaO_{2}-C\prime \prime }{CaO_{2}-CvO_{2}}$$ (11)

where C′ and C′′ are defined:

$$C\prime =\alpha O_{2}\cdot \frac{PaO_{2}+PvO_{2}}{2}+Hb\cdot {\bar{S}}_{ODC}\;$$ (12)

$$C\prime \prime =\alpha O_{2}\cdot {\bar{P}}_{ODC}+Hb\cdot \frac{SaO_{2}+SvO_{2}}{2}\;$$ (13)

Here ${\bar{S}}_{ODC}$ and ${\bar{P}}_{ODC}$ are the mean oxygen saturation and tension measured on the ODC, which are calculated by integration of the Hills equations from the arterial inlet to venous outlet.

$${\bar{S}}_{ODC}=\frac{1}{PaO_{2}-PvO_{2}}\cdot \overset{PaO_{2}}{\underset{PvO_{2}}{\int }}{\left(1+{\left(\frac{P_{50cap}}{PcapO_{2}}\right)}^{h}\right)}^{-1}\;{\text{dPcapO}}_{2}$$ (14)

$${\bar{P}}_{ODC}=\frac{1}{SaO_{2}-SvO_{2}}\overset{SaO_{2}}{\underset{SvO_{2}}{\int }}P_{50cap}\cdot {\left(\frac{ScapO_{2}}{1-ScapO_{2}}\right)}^{\frac{1}{h_{cap}}}\;{\text{dScapO}}_{2}$$ (15)

### Statistics

All statistical analyses were performed using R statistical software version 4.1.1 (R Project for Statistical Computing) within RStudio statistical software version 1.4.1717 (RStudio). Normality of data was confirmed by normality plots and homogeneity of variance was tested using the Bartlett test and data was collected in an independent manner. Changes from rest to KEE were analysed using a paired T-test. A one-way analysis of variance (ANOVA) was used to detect differences between groups and if the F test was significant (*p* <0.05), we used a Tukey’s *post-hoc* test for all group comparison combinations. Data are presented as mean (standard deviation), and differences are presented as mean. Significance was established at *p* <0.05. Effect sizes were calculated by Cohen’s D method.

## Results

Baseline characteristics are presented in Table 1. The V̇O_2_peak in the COPD group was similar to that of the CHF group, but COPD showed lower V̇O_2_peak compared to controls and there was no difference between CHF and controls The COPD and CHF group had similar 6MWD that were lower than in controls. The 10W workload during KEE comprised a similar percentage of maximal workload in the three groups (COPD: 33 (4) vs. CHF: 38 (23) vs. controls: 26 (7) %.

### Haemodynamics

At rest, there were no differences in MAP (COPD: 110 (14) vs. CHF: 103 (8) vs. controls: 113 (11) mmHg; *p*=0.23), LVC (COPD: 2.3 (1.1) vs. CHF: 1.8 (0.5) vs. controls: 1.9 (0.2) ml/(min*mmHg); *p*=0.25) or Q̇_leg_ (COPD: 222 (112) vs. CHF: 166 (43) vs. controls: 178 (22) ml/min; *p*=0.27) between the three groups.

**FIGURE 1:**
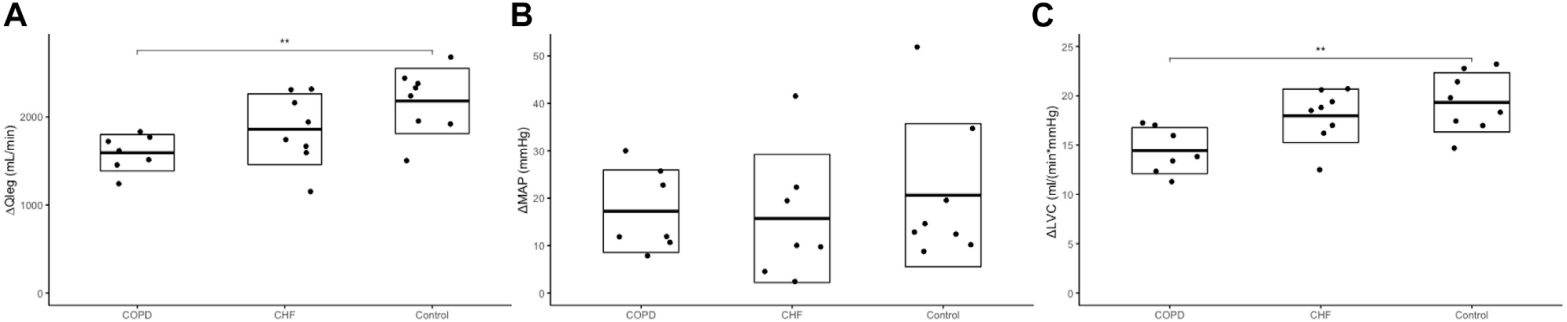
Haemodynamic responses. Panel **(A)** shows the change (Δ) in leg blood flow (Q_leg_) from rest to one leg knee-extensor exercise at 10 W, panel **(B)** change in mean arterial pressure (MAP) and panel **(C)** change in leg vascular conductance (LVC). One-way ANOVA, with Tukey’s honest test was used to detect differences between groups marked with a *= (*p* <0.05) or **= (*p* <0.01). Dot plots with boxes showing mean and standard deviation with exact values for each participant. Chronic heart failure (CHF) participants n=8, Chronic obstructive pulmonary disease (COPD) participants n = 7, healthy control *n* = 8.

During exercise, the Q̇_leg_, MAP and LVC all increased within both COPD, CHF, and controls (P<0.05). The change (Δ) from rest to exercise Q̇_leg_ responses were similar in COPD vs. CHF, lower in COPD vs. controls, but similar in CHF vs. controls (Figure 1). There were no differences in ΔMAP between groups. Therefore, ΔLVC responses during KEE in COPD vs. CHF were not different, lower in COPD vs. controls, and similar in CHF vs. controls.

### Skeletal muscle oxygen consumption and microvascular oxygenation

**FIGURE 2:**
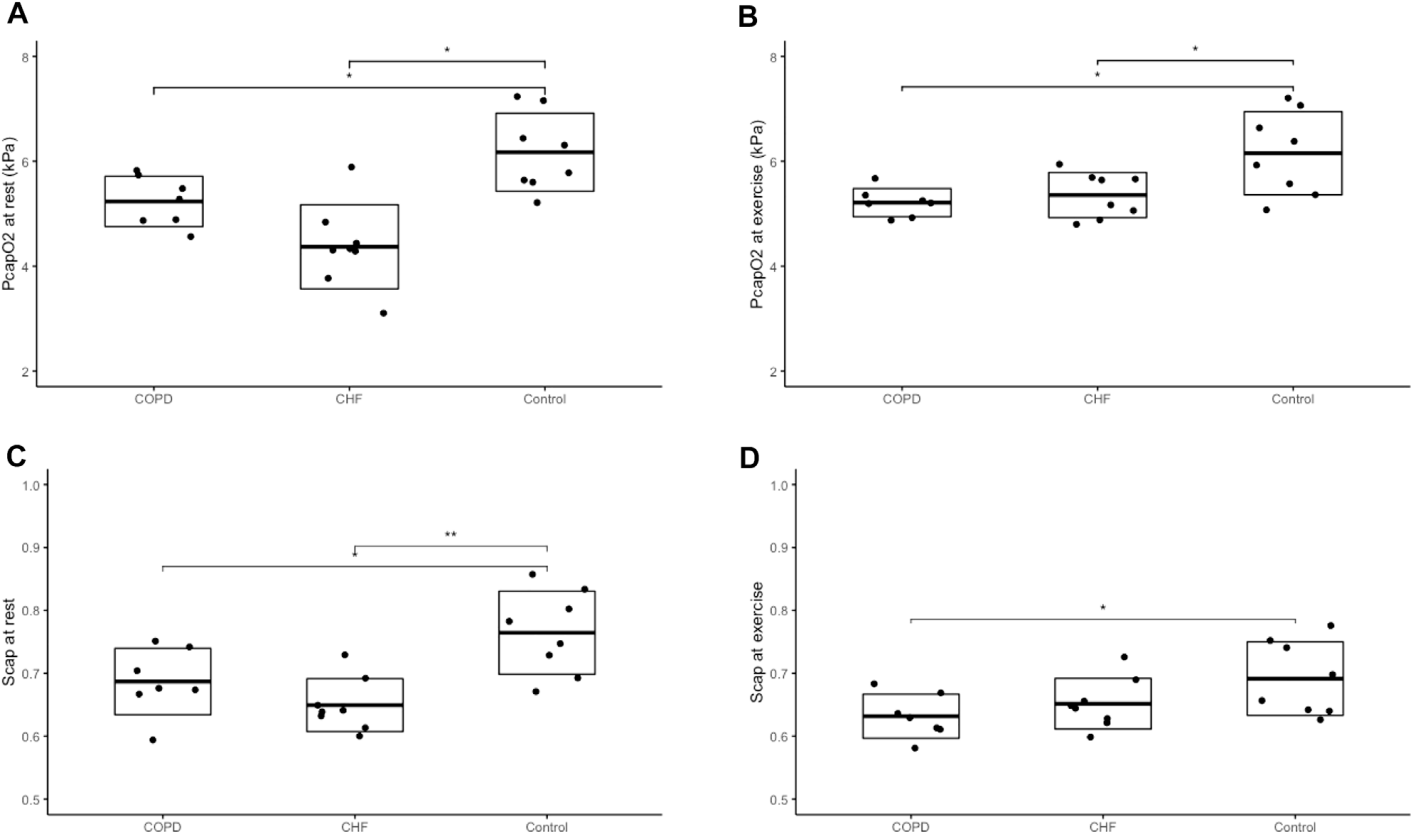
Capillary oxygen tension and saturation Panel **(A)** shows the capillary oxygen tension (PcapO_2_) at rest, panel **(B)** the capillary oxygen tension during one leg knee-extensor exercise, panel **(C)** the oxygen saturation (Scap) at rest and panel **(D)** the oxygen saturation during one leg knee-extensor exercise. We performed a one-way ANOVA and if significant, a Tukey’s honest significant difference test was used to analyse the differences between groups. Difference between groups marked with a *= (*p* <0.05), **= (*p* <0.01), ***= (*p* <0.001). Dot plots with boxes showing mean and standard deviation with exact values for each participant, marked with a black dot. Chronic heart failure (CHF) participants n=8, Chronic obstructive pulmonary disease (COPD) participants n = 7, healthy control n = 8.

Resting SaO_2_ and PaO_2_ were lower in the COPD group compared to the control group (Table 2). We found no differences in DelO_2_ between the three groups at rest (COPD: 2.1 (1.1) vs. CHF: 1.4 (0.4) vs. controls: 1.5 (0.1) mmol O_2_/min; *p*=0.2). Likewise, there was no difference in V̇O_2SM_ between COPD and CHF at rest (COPD: 1.1 (0.2) vs. CHF: 0.9 (0.3) mmol O_2_/min, (*p*=0.57). Resting V̇O_2SM_ was higher in COPD compared to controls (COPD: 1.1 (0.2) vs. controls: 0.7 (0.2) mmol O_2_/min; *p*=0.03), with no difference between CHF and controls groups. $\bar{P}capO_{2}$ and $\bar{S}capO_{2}$ were lower in both COPD and CHF compared to controls at rest (Figure 2).

**TABLE 2:** Blood gas values.

	COPD		CHF		Control	
	Rest	KEE	Rest	KEE	Rest	KEE
PaO_2_ (kPa)	10.3 (1.1) #	11.1 (1.1) #	11.1 (1.7)	12.0 (1.6)	12.0 (1.2)	14.5 (2.9) *
SaO_2_	96 (0.02) #	96 (0.01) #	97 (0.01)	97 (0.01)	97 (0.01)	97 (0.01)
CaO_2_ (mM)	8.8 (0.7)	9.0 (0.6)	8.4 (0.7)	8.5 (0.7)	8.6 (0.6)	8.7 (0.6) *
CvO_2_ (mM)	3.8 (1.1)	2.8 (0.7) *	2.9 (0.8) #	2.6 (0.8)	4.8 (1.1)	3.5 (0.8) *
PaCO_2_ (kPa)	4.4 (0.5)	4.75 (0.4)	4.68 (0.4)	4.7 (0.6)	4.6 (0.4)	4.6 (0.6)
pH (units)	7.43 (0.03)	7.41 (0.02)	7.41 (0.02)	7.40 (0.03) *	7.42 (0.02)	7.41 (0.01)
Lactate (mM)	0.7 (0.3)	1.5 (0.2) *	0.9 (0.4)	3.1 (2) *	0.6 (0.2)	2.1 (1) *

Data in means (standard deviation). Blood gas variables at rest and during one leg knee-extensor exercise (KEE) at 10 W. We performed a one-way ANOVA, and if significant, a Tukey’s honest significant difference was used to analyse the differences between groups. Comparison between rest and exercise was performed using a paired t-test. * = Different from baseline (*p* <0.05). # = Different from control group (*p* <0.05). *n*=8 for resting COPD, values, n=7 for KEE, values. N=8 for both rest and KEE, values for CHF, and controls. *Abbreviations:* PaO_2_: arterial oxygen partial pressure SaO_2_: arterial oxygen saturation. Hgb: Haemoglobin. CaO_2_: arterial oxygen content. CvO_2_: venous oxygen content. Pa_CO2_: arterial carbon dioxide partial pressure.

**FIGURE 3:**
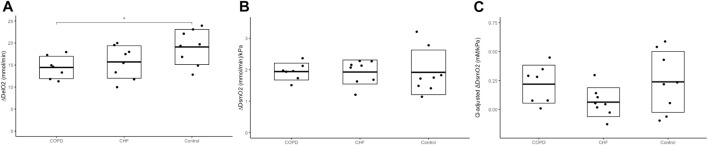
Skeletal muscle oxygen delivery and conductance Panel **(A)** shows the change (Δ) in O_2_ delivery (ΔDelO_2_) from rest to one leg knee-extensor exercise at 10 W, panel **(B)** change in skeletal muscle oxygen conductance (D_SM_O_2_) and panel **(C)** change in flow adjusted skeletal muscle oxygen conductance (Q̇-adjusted D_SM_O_2_). We performed a one-way ANOVA and if significant, a Tukey’s honest significant difference test was used to analyse the differences between groups. Difference between groups marked with a *= (*p* <0.05) and **= (*p* <0.01). Dot plots with boxes showing mean and standard deviation with exact values for each participant, marked with a black dot. Chronic heart failure (CHF) participants n = 8, Chronic obstructive pulmonary disease (COPD) participants n = 7, healthy control n = 8.

Exercising SaO_2_ and PaO_2_ were also lower in COPD vs. controls. We found no differences in DelO_2_ during KEE (COPD: 16.5 (3.3) vs. CHF: 17.0 (3.7) vs. controls: 20.6 (3.1) mmol O_2_/min; *p*=0.2). The ΔDelO_2_ was higher in controls compared to COPD in response to exercise (Figure 3), but similar to CHF, with no difference between COPD and CHF. The $\bar{S}capO_{2}$ was lower in COPD than in controls during exercise, but not between the other groups (CHF vs. COPD or control vs. CHF)**.** Despite these differences, the V̇O_2SM_ increased to almost similar absolute levels in all three groups during KEE (COPD: 11.2 (1.6) vs. CHF: 11.0 (1.8) vs. controls: 12.1 (2.8) mmol O_2_/min; *p*=0.57).

### Skeletal muscle oxygen conductance

At rest, there were no difference in D_SM_O_2_ between COPD and CHF (COPD: 0.21 (0.08) vs. CHF 0.16 (0.04) mmol/min/kPa; *p*=0.2), but D_SM_O_2_ was higher in both COPD and CHF than controls (0.11 (0.04) mmol/min/kPa, *p*=0.005). After Q̇-adjustment, there was still no difference in resting D_SM_O_2_ between COPD and CHF, and likewise, both groups showed higher Q̇-adjusted D_SM_O_2_ than controls (COPD: 0.97 (0.23) vs. controls 0.63 (0.24) mM/kPa, *p*=0.02; CHF 0.98 (0.11) mM/kPa vs. controls, *p*=0.001)

In response to exercise, D_SM_O_2_ increased in all groups (*p* <0.05) to reach similar values (COPD: 2.15 (0.3) vs. CHF: 2.1 (0.4) vs. controls: 2.0 (0.7) mmol/min/kPa. In contrast to the unadjusted D_SM_O_2_ response to exercise, the Q̇-adjusted D_SM_O_2_ was significantly increased in COPD (*p*=0.01) and controls (*p*=0.03), but not in the CHF group (*p*=0.2). However, we found no difference between COPD and CHF in Q̇-adjusted D_SM_O_2_ during exercise (COPD: 1.19 (0.11) vs. CHF: 1.00 (0.18) mM/kPa; *p*=0.24), although Q̇-adjusted D_SM_O_2_ was higher in COPD compared to controls (COPD vs. controls: 0.87 (0.28) mM/kPa, *p*=0.02), and similar in CHF and controls.

## Discussion

We studied leg muscle convective and diffusive O_2_ transport during small muscle mass exercise in COPD, CHF and healthy controls. There were no significant differences between COPD and CHF but we did observe disease-specific macro- and microvascular responses to exercise compared to the healthy condition. We found that while the skeletal muscle O_2_ delivery during exercise was lower in COPD, the microvasculature responded with an enhanced Q̇-adjusted D_SM_O_2_ or perhaps *vice versa*, the O_2_ delivery adapted to the increased muscle diffusion capacity seen at rest. This response was not observed in CHF, where Q̇-adjusted D_SM_O_2_ was unresponsive to exercise, despite high resting D_SM_O_2_ (Q-adjusted and unadjusted) values compared to controls. Likewise, CHF patients were not able to decrease C_v_O_2_ in response to exercise, as observed in COPD and controls, suggesting impaired O_2_ extraction during exercise. The LVC responses to exercise in CHF was similar to controls, indicating that the macrovascular response was preserved, while impaired in COPD. Nonetheless, both groups reached normal V̇O_2SM_ values as expected during submaximal small muscle mass exercise at similar work loads. These data suggest that enhanced diffusive O_2_ transport capacity of the working muscles in COPD compensates for the attenuated convective O_2_ transport, while the convective response to exercise in CHF may downregulate muscle diffusive O_2_ transport capacity.

### Microvascular (mal)adaptation in chronic obstructive pulmonary disease

D_SM_O_2_ is an estimate of oxygen transport capacity from the capillary to the mitochondria (Østergaard, 2020). It comprises a convective component and a diffusive component, of which the latter depends on the total capillary wall area available for diffusion during red blood cell passage, reflecting the total number of perfused capillaries and their length, the capillary flow distribution, spatial distribution of capillaries and potential structural changes in the diffusion membrane (Wagner, 2000; Poole, 2019; Nyberg and Jones, 2022). The relative interdependence of convection and diffusion is difficult to establish during human muscle contractions. If, one is impaired, the other might compensate to some degree. Likewise, low convective response to small muscle mass exercise in COPD is not a universal observation in previous studies. Indeed, although some studies like us have found lower convective responses to exercise in severe COPD compared to controls (Brønstad et al., 2012; Broxterman et al., 2021), others reported either similar (Rossman et al., 2015) or even higher Q̇_leg_ during exercise (Richardson et al., 2004) in moderate and severe COPD, respectively. These somewhat divergent findings are most likely due to the large difference between the control groups of the abovementioned studies. In our patients with moderate-to-severe COPD (FEV_1_ ∼50% of predicted), diffusive oxygen transport was augmented to enhance skeletal muscle O_2_ uptake despite an impaired blood flow response. In more severe COPD (FEV_1_ ∼30% of predicted), the diffusive response during KEE has previously been reported to be impaired, appeared to be specifically enhanced by 8 weeks of KEE training (Broxterman et al., 2021). Together with our findings, this indicates that enhanced diffusive oxygen transport has the potential to counter the impact of an insufficient blood flow response on V̇O_2_peak and that its gradual decline with disease severity is potentially reversible, i.e., by exercise training.

### Microvascular (mal)adaptation in chronic heart failure

In our CHF patients, we found that the diffusive response to KEE seemed to be limited, which is in line with findings in some previous studies (Esposito et al., 2011). Thus, reductions in skeletal muscle diffusive oxygen transport have been reported in patients with CHF NYHA II-III, and of note, Esposito et al (2011) observed that exercise training by KEE over an eight-week period improved the diffusive component in the CHF patients to the same level as the healthy control group at baseline. Furthermore, they observed an augmented capillarization of the quadriceps muscle in parallel with improved peripheral exercise capacity after isolated leg muscle training for 8 weeks, but the cardiac output remained unchanged, suggesting that increases in V̇O_2SM_ can be obtained without “central” improvements (Esposito et al., 2011). The same group performed another study where the control group was better matched on physical fitness and found no significant differences in haemodynamics and muscle metabolism, comparing CHF (NYHA II-III) and sedentary controls (Esposito et al., 2015). The convective response to small muscle exercise has been reported to be impaired in patients with CHF (Barrett-O’Keefe et al., 2014, 2019), while others have found that if less than 4 kg of muscle is activated the convective capacity was similar to healthy individuals (Magnusson et al., 1997). Although the numerical values in the present study showed a tendency towards lower convective responses in CHF during KEE, this was not significant when all groups were analysed in parallel and after statistical adjustment (Figure 1). A possible explanation for the divergent results across previous studies and in relation to our COPD group could be the pharmacological management of CHF patients. For example, ACE-inhibitors commonly prescribed to CHF patients affects cardiovascular regulation by relaxation of the vascular smooth muscle of resistance arterioles (Anderson et al., 2000; Radenković et al., 2019). Over the past 30 years, during which the abovementioned studies have been conducted, there has been a decrease in the use of positive inotropes such as digoxin. The interaction between these drugs and exercise must be considered because they affect the autonomic control of cardiovascular function (Watanabe, 1985; Gheorghiade et al., 1989; Heidenreich et al., 1997; van der Harst and de Boer, 2010) and vasodilatory capacity in skeletal muscle (Karsh and Bullock, 1964) which are pivotal to the peripheral adaptations to exercise.

While a reduced blood flow response to exercise in COPD was observed, and potentially also play a role in CHF, the main difference between the two disease categories was that diffusive oxygen transport was either near-maximal already at rest, or simply unresponsive to exercise in CHF, whilst augmented in skeletal muscle in COPD.

### Study limitations

This study was performed on data from earlier studies with a new methodology. The mechanisms underlying exercise intolerance are complex and often multifactorial in both COPD and CHF patients. The pathophysiological factors that contribute are ventilatory limitations, gas exchange deficits, reduced cardiac function, and limb muscle dysfunction, and any combination might dominate the phenotype. There were no differences in ΔMAP between groups to suggest a heightened exercise pressor reflex activation, but CHF and COPD patients have been associated with sensitization of the exercise pressor reflex (Smith et al., 2005), which may contribute to their exercise intolerance (Gagnon et al.; Amann et al., 2014; Smith et al., 2020). We cannot exclude that a difference in exercise pressor reflex activation may have influenced our results, as inhibition of lower limb muscle mechano- and metabo-sensitive afferents has shown to increase exercise tolerance in CHF and COPD, perhaps through increased O_2_ delivery to the working muscle (Amann et al., 2014; Smith et al., 2020). Another challenge for this kind of study is sample size which is relatively small because of the invasive nature of these experiments, and results may not be extrapolated to all patients with COPD or CHF. The present findings were based on the KEE model, but it remains uncertain whether the findings also apply during more intense exercise involving a larger muscle mass or other muscle groups. It is difficult to find “matched” individuals that are healthy, but still has a very low level of physical activity, making it problematic to compare groups if there are fundamental differences in physical fitness. The effect sizes of these variables ranged from small to large, due to the small number of included patients and the large standard deviations.

### Clinical implications

Exercise training, often being conducted on a two-legged bike ergometer, is known to be one of the most effective treatments for improving quality of life and to increase exercise capacity in both CHF and COPD patients, mostly thought to be caused by “central” improvement (Hunt et al., 2005; Stav et al., 2009; Mccarthy et al., 2015; Spruit et al., 2016; Paneroni et al., 2017; Gao et al., 2021). Based on our data and the current literature it seems plausible that there is a potential for improvements of the peripheral oxygen conductance in skeletal muscle in COPD and CHF patients even if cardio-pulmonary function stays unaltered.

## Conclusion

Disease-specific factors may play a role in peripheral exercise capacity in patients with COPD and CHF. Thus, low convective O_2_ transport to contracting muscle seemed to predominate the peripheral exercise limitation in COPD during small muscle mass exercise, whereas muscle diffusive O_2_ transport was unresponsive to exercise in CHF.

## Data Availability

The raw data supporting the conclusions of this article will be made available by the authors, without undue reservation.

## References

[B1] Amann M. Venturelli M. Ives S. J. Morgan D. E. Gmelch B. Witman M. A. H. (2014). Group iii/iv muscle afferents impair limb blood in patients with chronic heart failure. Int. J. Cardiol. 174, 368–375. 10.1016/j.ijcard.2014.04.157 24794967 PMC4075285

[B2] Anderson T. J. Elstein E. Haber H. Charbonneau F. (2000). Comparative study of ACE-inhibition, angiotensin II antagonism, and calcium channel blockade on flow-mediated vasodilation in patients with coronary disease (BANFF study). J. Am. Coll. Cardiol. 35, 60–66. 10.1016/S0735-1097(99)00537-9 10636260

[B3] Ausín P. Martínez-Llorens J. Sabaté-Bresco M. Casadevall C. Barreiro E. Gea J. (2017). Sex differences in function and structure of the quadriceps muscle in chronic obstructive pulmonary disease patients. Chron. Respir. Dis. 14, 127–139. 10.1177/1479972316674412 27923983 PMC5720222

[B4] Barrett-O'Keefe Z. Lee J. F. Berbert A. Witman M. A. H. Nativi-Nicolau J. Stehlik J. (2014). Hemodynamic responses to small muscle mass exercise in heart failure patients with reduced ejection fraction. Am. J. Physiology-Heart Circulatory Physiology 307, H1512–H1520. 10.1152/ajpheart.00527.2014 PMC4280163 25260608

[B5] Barrett-O’Keefe Z. Lee J. F. Ives S. J. Trinity J. D. Witman M. A. H. Rossman M. J. (2019). α-Adrenergic receptor regulation of skeletal muscle blood flow during exercise in heart failure patients with reduced ejection fraction. Am. J. Physiology-Regulatory, Integr. Comp. Physiology 316, R512–R524. 10.1152/ajpregu.00345.2018 PMC6589600 30789790

[B6] Brønstad E. Rognmo O. Tjonna A. E. Dedichen H. H. Kirkeby-Garstad I. Håberg A. K. (2012). High-intensity knee extensor training restores skeletal muscle function in COPD patients. Eur. Respir. J. 40, 1130–1136. 10.1183/09031936.00193411 22408206

[B7] Broxterman R. M. Wagner P. D. Richardson R. S. (2021). Exercise training in COPD: Muscle O_2_ transport plasticity. Eur. Respir. J. 58, 2004146. 10.1183/13993003.04146-2020 33446612

[B8] Buber J. Robertson H. T. (2022). Cardiopulmonary exercise testing for heart failure: Pathophysiology and predictive markers. Heart. 2021, 319617. 10.1136/heartjnl-2021-319617 35410893

[B9] Dahl R. H. Taudorf S. Bailey D. M. Møller K. Berg R. M. G. (2020). A method for modelling the oxyhaemoglobin dissociation curve at the level of the cerebral capillary in humans. Exp. Physiol. 105, 1063–1070. 10.1113/EP088615 32436618

[B10] Dempsey J. A. (2019). Respiratory determinants of exercise limitation. Clin. Chest Med. 40, 331–342. 10.1016/j.ccm.2019.02.002 31078213 PMC6512838

[B11] Esposito F. Reese V. Shabetai R. Wagner P. D. Richardson R. S. (2011). Isolated quadriceps training increases maximal exercise capacity in chronic heart failure. J. Am. Coll. Cardiol. 58, 1353–1362. 10.1016/j.jacc.2011.06.025 21920265 PMC3180857

[B12] Esposito F. Wagner P. D. Richardson R. S. (2015). Incremental large and small muscle mass exercise in patients with heart failure: Evidence of preserved peripheral haemodynamics and metabolism. Acta Physiol. 213, 688–699. 10.1111/apha.12423 PMC4540072 25393513

[B13] Gagnon P. Bussières J. S. Ribeiro F. Gagnon S. L. Saey D. Gagné N. (2012). Influences of spinal anesthesia on exercise tolerance in patients with chronic obstructive pulmonary disease. Am. J. Respir. Crit. Care Med. 186, 606–615. 10.1164/rccm.201203-0404OC 22822019

[B14] Gao M. Huang Y. Wang Q. Liu K. Sun G. (2021). Effects of high-intensity interval training on pulmonary function and exercise capacity in individuals with chronic obstructive pulmonary disease: A meta-analysis and systematic review. Adv. Ther. 39, 94–116. 10.1007/s12325-021-01920-6 34792785

[B15] Gheorghiade M. Hall V. Lakier J. B. Goldstein S. (1989). Comparative hemodynamic and neurohormonal effects of intravenous captopril and digoxin and their combinations in patients with severe heart failure. J. Am. Coll. Cardiol. 13, 134–142. 10.1016/0735-1097(89)90561-5 2562844

[B16] Heidenreich P. A. Lee T. T. Massie B. M. (1997). Effect of beta-blockade on mortality in patients with heart failure: A meta-analysis of randomized clinical trials 11All editorial decisions for this article, including selection of referees, were made by a guest editor. This policy applies to all articles with authors from the university of California san francisco. J. Am. Coll. Cardiol. 30, 27–34. 10.1016/S0735-1097(97)00104-6 9207617

[B17] Houstis N. E. Eisman A. S. Pappagianopoulos P. P. Wooster L. Bailey C. S. Wagner P. D. (2018). Exercise intolerance in heart failure with preserved ejection fraction. Circulation 137, 148–161. 10.1161/CIRCULATIONAHA.117.029058 28993402 PMC5760316

[B18] Hunt S. A. Abraham W. T. Chin M. H. Feldman A. M. Francis G. S. Ganiats T. G. (2005). ACC/AHA 2005 guideline update for the diagnosis and management of chronic heart failure in the adult. J. Am. Coll. Cardiol. 46, e1–e82. 10.1016/j.jacc.2005.08.022 16168273

[B19] Iepsen U. W. Munch G. W. Rugbjerg M. Ryrsø C. K. Secher N. H. Hellsten Y. (2017). Leg blood flow is impaired during small muscle mass exercise in patients with COPD. J. Appl. Physiology 123, 624–631. 10.1152/japplphysiol.00178.2017 28729387

[B20] Iepsen U. W. Munch G. W. Ryrsø C. K. Secher N. H. Lange P. Thaning P. (2018). Muscle α-adrenergic responsiveness during exercise and ATP-induced vasodilation in chronic obstructive pulmonary disease patients. Am. J. Physiology-Heart Circulatory Physiology 314, H180–H187. 10.1152/ajpheart.00398.2017 29030339

[B21] Karsh R. B. Bullock F. A. (1964). Studies on digitalis. X. Effects of ouabain on forearm vas-cular resistance and venous tone in normal sub-jects and in patients in heart failure. J. Clin. Invest. 43, 532–543. 10.1172/JCI104939 14135505 PMC441947

[B22] Keller-Ross M. L. Larson M. Johnson B. D. (2019). Skeletal muscle fatigability in heart failure. Front. Physiol. 10, 1–8. 10.3389/fphys.2019.00129 30846944 PMC6393404

[B23] Magnusson G. Kaijser L. Sylvén C. Karlberg K. E. Isberg B. Saltin B. (1997). Peak skeletal muscle perfusion is maintained in patients with chronic heart failure when only a small muscle mass is exercised. Cardiovasc. Res. 33, 297–306. 10.1016/S0008-6363(96)00249-0 9074693

[B24] Maltais F. Decramer M. Casaburi R. Barreiro E. Burelle Y. Debigaré R. (2014). An official American thoracic society/european respiratory society statement: Update on limb muscle dysfunction in chronic obstructive pulmonary disease. Am. J. Respir. Crit. Care Med. 189, e15–e62. 10.1164/rccm.201402-0373ST 24787074 PMC4098112

[B25] McCarthy B. Casey D. Devane D. Murphy K. Murphy E. Lacasse Y. (2015). Pulmonary rehabilitation for chronic obstructive pulmonary disease. Cochrane Database Syst. Rev. 23, CD003793. 10.1002/14651858.CD003793.pub3 PMC10008021 25705944

[B26] Mortensen S. P. Saltin B. (2014). Regulation of the skeletal muscle blood flow in humans. Exp. Physiol. 99, 1552–1558. 10.1113/expphysiol.2014.081620 25192730

[B27] Mortensen S. P. Wewer U. Sp M. Damsgaard R. Saltin B. (2010). Regulation of skeletal muscle blood flow and central hemodynamics in exercising humans.

[B52] Munch G. W. Iepsen U.W. Ryrsø C. K. Rosenmeier J. B. Pedersen B. K. Mortensen S. P. (2018). Effect of 6 weeks of high-intensity one-legged cycling on functional sympatholysis and ATP signaling in patients with heart failure. Am. J. Physiology-Heart Circulatory Physiology 314, H616–H626. 10.1152/ajpheart.00379.2017 29167117

[B29] Nyberg M. Jones A. M. (2022). Matching of O_2_ utilization and O_2_ delivery in contracting skeletal muscle in health, aging, and heart failure. Front. Physiol. 13, 1–15. 10.3389/fphys.2022.898395 PMC9237395 35774284

[B30] Østergaard L. (2020). Blood flow, capillary transit times, and tissue oxygenation: The centennial of capillary recruitment. J. Appl. Physiology 129, 1413–1421. 10.1152/JAPPLPHYSIOL.00537.2020 33031017

[B31] Paneroni M. Simonelli C. Vitacca M. Ambrosino N. (2017). Aerobic exercise training in very severe chronic obstructive pulmonary disease. Am. J. Phys. Med. Rehabil. 96, 541–548. 10.1097/PHM.0000000000000667 28099192

[B32] Pittman R. N. (2000). Oxygen supply to contracting skeletal muscle at the microcirculatory level: Diffusion vs. convection. Acta Physiol. Scand. 168, 593–602. 10.1046/J.1365-201X.2000.00710.X 10759595

[B33] Poole D. C. (2019). Edward F. Adolph distinguished lecture. Contemporary model of muscle microcirculation: Gateway to function and dysfunction. J. Appl. Physiology 127, 1012–1033. 10.1152/japplphysiol.00013.2019 PMC6850982 31095460

[B34] Poole D. C. Musch T. I. Colburn T. D. (2022). Oxygen flux from capillary to mitochondria: Integration of contemporary discoveries. Eur. J. Appl. Physiol. 122, 7–28. 10.1007/s00421-021-04854-7 34940908 PMC8890444

[B35] Poole D. C. Richardson R. S. Haykowsky M. J. Hirai D. M. Musch T. I. (2018). Exercise limitations in heart failure with reduced and preserved ejection fraction. J. Appl. Physiology 124, 208–224. 10.1152/japplphysiol.00747.2017 PMC5866447 29051336

[B36] Radenković M. Stojanović M. Prostran M. (2019). Calcium channel blockers in restoration of endothelial function: Systematic review and meta-analysis of randomized controlled trials. Cmc 26, 5579–5595. 10.2174/0929867325666180713144806 30009701

[B37] Richardson R. S. Leek B. T. Gavin T. P. Haseler L. J. Mudaliar S. R. D. Henry R. (2004). Reduced mechanical efficiency in chronic obstructive pulmonary disease but normal peak Vo2 with small muscle mass exercise. Am. J. Respir. Crit. Care Med. 169, 89–96. 10.1164/rccm.200305-627oc 14500263

[B38] Rossman M. J. Trinity J. D. Garten R. S. Ives S. J. Conklin J. D. Barrett-O'Keefe Z. (2015). Oral antioxidants improve leg blood flow during exercise in patients with chronic obstructive pulmonary disease. Am. J. Physiology-Heart Circulatory Physiology 309, H977–H985. 10.1152/ajpheart.00184.2015 PMC4591404 26188020

[B39] Saltin B. (1985). Maximal perfusion of skeletal muscle in man. J. Physiol. 366, 233–249. 10.1113/jphysiol.1985.sp015794 4057091 PMC1193029

[B40] Shibata M. Ichioka S. Togawa T. Kamiya A. (2006). Arterioles' contribution to oxygen supply to the skeletal muscles at rest. Eur. J. Appl. Physiol. 97, 327–331. 10.1007/s00421-006-0200-2 16770469

[B28] Smith J. R. Joyner M. J. Curry T. B. Borlaug B. A. Keller-Ross M. L. Van Iterson E. H. (2020). Locomotor muscle group III/IV afferents constrain 1057 stroke volume and contribute to exercise intolerance in human heart failure. J. Physiol. 598, 5379–5390. 10.1113/jp280333 32886795 PMC10039366

[B41] Smith J. R. Joyner M. J. Curry T. B. Borlaug B. A. Keller‐Ross M. L. Van Iterson E. H. (2020). Locomotor muscle group III/IV afferents constrain stroke volume and contribute to exercise intolerance in human heart failure. J. Physiol. 598, 5379–5390. 10.1113/jp280333 32886795 PMC10039366

[B42] Smith S. A. Mitchell J. H. Naseem H. R. Garry M. G. (2005). Mechanoreflex mediates the exaggerated exercise pressor reflex in heart failure. Circulation 112, 2293–2300. 10.1161/CIRCULATIONAHA.105.566745 16216976

[B43] Spruit M. A. Burtin C. De Boever P. Langer D. Vogiatzis I. Wouters E. F. M. (2016). COPD and exercise: Does it make a difference? Breathe 12, e38–e49. 10.1183/20734735.003916 27408645 PMC4933612

[B44] Stav D. Raz M. Shpirer I. (2009). Three years of pulmonary rehabilitation: Inhibit the decline in airflow obstruction, improves exercise endurance time, and body-mass index, in chronic obstructive pulmonary disease. BMC Pulm. Med. 9, 26. 10.1186/1471-2466-9-26 19480709 PMC2693131

[B45] van der Harst P. de Boer R. A. (2010). Statins in the treatment of heart failure. Circ. Heart Fail. 3, 462–464. 10.1161/CIRCHEARTFAILURE.110.956342 20484198

[B46] Vestbo J. Hurd S. S. Agustí A. G. Jones P. W. Vogelmeier C. Anzueto A. (2013). Global strategy for the diagnosis, management, and prevention of chronic obstructive pulmonary disease. Am. J. Respir. Crit. Care Med. 187, 347–365. 10.1164/rccm.201204-0596PP 22878278

[B47] Wagner P. D. (1996). Determinants of maximal oxygen transport and utilization. Annu. Rev. Physiol. 58, 21–50. 10.1146/annurev.ph.58.030196.000321 8815793

[B48] Wagner P. D. (2000). Diffusive resistance to O_2_ transport in muscle. Acta Physiol. Scand. 168, 609–614. 10.1046/j.1365-201X.2000.00712.x 10759597

[B49] Watanabe A. M. (1985). Digitalis and the autonomic nervous system. J. Am. Coll. Cardiol. 5, 35A–42A. 10.1016/S0735-1097(85)80461-7 3886751

[B50] Zizola C. Schulze P. C. (2013). Metabolic and structural impairment of skeletal muscle in heart failure. Heart Fail Rev. 18, 623–630. 10.1007/s10741-012-9353-8 23065040 PMC3784612

