# Erratum: Regulation of the microvasculature during small muscle mass exercise in chronic obstructive pulmonary disease vs. chronic heart failure

**DOI:** 10.3389/fphys.2022.1062951

**Published:** 2022-10-31

**Authors:** Frontiers Production Office

**Affiliations:** Frontiers Media SA, Lausanne, Switzerland

**Keywords:** COPD, chronic obstructive pulmonary disease, heart failiure, knee extensor exercise, capillary recruitment, exercise capacity

Due to a production error, one of the in-text citations under the sub-heading “Participants” was changed from Munch et al. (2018) to Smith et al. (2020).

The publisher apologises for this mistake. The original article has been updated.

